# Comparative immune responses after vaccination with the formulated inactivated African horse sickness vaccine serotype 1 between naïve horses and pretreated horses with the live-attenuated African horse sickness vaccine

**DOI:** 10.14202/vetworld.2022.2365-2375

**Published:** 2022-10-07

**Authors:** Narongsak Chaiyabutr, Suphot Wattanaphansak, Rachod Tantilerdcharoen, Surasak Akesowan, Suraseha Ouisuwan, Darm Naraporn

**Affiliations:** 1 Department of Research and Development, Queen Saovabha Memorial Institute, Thai Red Cross Society, Bangkok, Thailand; 2 Department of Physiology, Faculty of Veterinary Science, Chulalongkorn University, Bangkok, Thailand; 3 Department of Veterinary Medicine, Faculty of Veterinary Science Chulalongkorn University, Bangkok, Thailand; 4 Veterinary Diagnostic Laboratory, Faculty of Veterinary Science Chulalongkorn University, Bangkok, Thailand; 5 Horse Farm and Laboratory Animal Breeding Centre, Queen Saovabha Memorial Institute, Thai Red Cross Society, Petchaburi, Thailand

**Keywords:** African horse sickness, inactivated vaccine, naïve young horse

## Abstract

**Background and Aim:**

African horse sickness (AHS) is a non-contagious, high mortality, and insect-borne disease caused by a double-stranded RNA virus from the genus *Orbivirus*. The study aimed to develop inactivated vaccines serotype 1 inactivated AHS vaccine (IAV) and to compare the effect of IAV on antibody responses in young naïve horses and adult horses pre-immunized with live-attenuated AHS virus (AHSV) serotypes 1, 3, and 4 live-attenuated vaccine (LAV).

**Materials and Methods:**

A total of 27 horses were vaccinated in two trials. Twelve AHS naïve young horses and 15 adult horses were divided into three groups of 4 and 5 horses each, respectively. Horses in control Group 1 were treated with phosphate-buffered saline. Horses in Group 2 were subcutaneously vaccinated with 2 mL of formulated IAV with 10% Gel 01™ (Seppic, France) on day 0 and horses in Group 3 were subcutaneously vaccinated with 2 mL of IAV on day 0 and a booster on day 28. The IAV vaccine was prepared by isolating the AHSV serotype 1 growing on Vero cells, 10× virus titer was concentrated by ultrafiltration and chemically killed by formalin, using 10% Gel 01™ as an adjuvant. Ethylenediaminetetraacetic acid blood samples were taken for hematology, blood biochemistry, and antibody titers using an immunoperoxidase monolayer assay on 158^th^ day post-vaccination.

**Results:**

Vaccination with IAV serotype 1 in adult horses pretreated with LAV increased antibody titers more than in young naïve vaccinated horses. The total leukocyte count and %neutrophils significantly increased, while %lymphocytes and %eosinophils significantly decreased on day 1 after vaccination; no local reactions were observed at the site of injection in any group. All biochemical and electrolyte analyte values were within the normal range after vaccination.

**Conclusion:**

The formulation of IAV serotype 1 using Gel 01™ as an adjuvant is safe and induces high antibody titers. This IAV formulation induced a high antibody response in horses without causing local reactions and mild systemic effects. However, AHS naïve horses still required ≥2 vaccinations and an annual booster vaccination to achieve high antibody titers.

## Introduction

African horse sickness (AHS) is a disease that causes high mortality rates of up to 95% for naïve horses and with potential for rapid spread [1]. The AHS outbreaks lead to huge economic losses by horse deaths, draft power reduction, and blockade of transportation and trade [2]. The AHS can result in four classical forms of the disease: Pulmonary, cardiac, mixed pulmonary and cardiac forms, and horse sickness fever [3, 4]. The causative agent, AHS virus (AHSV), genus *Orbivirus*, Reoviridae family, is a multi-component linear double-stranded RNA, that causes different forms of disease ranging from mild fever to subacute and acute infections [3, 5]. The AHSV is transmitted by biting midges (*Culicoides* spp.); the disease is most prevalent in areas where these vectors are most abundant. The hot-humid climatic conditions of Thailand favor the rapid expansion of *Culicoides* biting midges for virus transmission associated with climate change [6].

AHS is usually endemic in sub-Saharan Africa, a controlled disease in South Africa, and periodically reported in India, Pakistan, and Southeast Asia. In 2020 [7], AHS caused the death of 550 horses in Northeast Thailand, while 20,000 were vaccinated using polyvalent live attenuated AHS vaccine serotypes 1, 3, and 4 (live-attenuated vaccine [LAV]; Onderstepoort Biological Products). Thailand shares its international boundaries with neighboring countries, including southwest China which has a hot and humid climate. The wide geographic distribution of Culicoides spp. in hot and humid climates would play a role as a vector in the transmission of AHSV among neighboring countries. The density of the horse population on the southern border of China is more than that in Thailand and neighboring countries. The prediction for an outbreak in a high-risk area, especially the southern part of China, may be the risk of reintroducing AHS into China [8]. On this basis, a protocol has proposed the establishment of an AHS-free zone in Thailand which is of great importance for the surveillance and control of AHSV spreading. At present, prevention and control are based on controlling disease-spreading insects and the use of multivalent cocktails of LAV vaccines. The major concern of multivalent LAV vaccines refers to residual virulence, reverse virulence, and reassortment, subsequently leading to virus spread by midges, reproduction losses, and virulent AHSV variants [1, 9]. Therefore, these factors need to be considered when using multivalent LAV vaccines in an area without prior history of AHS outbreaks. Inactivated AHS vaccine (IAV) may be considered safe in this context, as they have the advantage of not containing a live and potentially dangerous agent. However, production may be expensive and multiple inoculations may be required to elicit and maintain high levels of protective immunity. It may also be difficult to ensure complete vaccine inactivation [10]. This explains why few IAVs are available on the market [11, 12]. To rapidly establish the AHSV serotype involved in the outbreak, a team of researchers at the Queen Saovabha Memorial Institute (QSMI) and Faculty of Veterinary Science, Chulalongkorn University, developed inactivated vaccines from serotypes 1 isolated from horse fatalities in Thailand. Our study hypothesized that in case of a recurring AHS epidemic in Thailand, a well-characterized vaccine manufactured using a standardized method should be available shortly by QSMI manufacturer(Thailand) and as soon as the responsible virus serotype has been identified.

Therefore, the study aimed to, first, determine the AHSV strain responsible for the emerging disease in Thailand in 2020 to develop a vaccine with formulated IAV containing serotypes 1 against AHSV; second, to compare the effect of IAV serotype 1 on the immune responses between 2–3-year-old adult horses pre-vaccination with LAV and 6–7-month-old AHS naïve young horses. This study aimed to provide a descriptive evaluation of serological monitoring of the vaccinated equine population against wild-type AHSV infection to quickly eradicate AHS in Thailand. Vaccination may lead to the production of safe and effective IAVs that protect equids against the disease before available new vaccines platform in the future, i.e. modern recombinant subunit AHS vaccines.

## Materials and Methods

### Ethical approval

All animal experiments were approved by the Animal Ethics Committee in accordance with Queen Saovabha Memorial Institute regulations and policies governing the care and use of laboratory animals. The study followed guidelines documented in ethical principles and guidelines of the National Research Council of Thailand for the care and the use of experimental animals. (Protocol No. QSMI-ACUC-07-2021).

### Study period and location

This study was divided into two parts; part 1 was performed for preliminary experiments in mice at Department of Veterinary Medicine, and Veterinary Diagnostic Laboratory, Faculty of Veterinary Science, Chulalongkorn University, Bangkok, from February 2021 to May 2021. Part 2 of this study was performed on horses for horse vaccination study at Horse Farm, QSMI, Phetchaburi Province, and horse blood samples were collected and processed at Department of Veterinary Medicine, Faculty of Veterinary Science, Chulalongkorn University, Bangkokfrom June 2021 to February 2022. The laboratory investigation of pathogenicity and vaccine efficacy study was conducted both in the Faculty of Veterinary Science, Chulalongkorn, and Horse Farm and Laboratory Animal Breeding Centre, QSMI, Thai Red Cross Society, Phetchaburi Province, Thailand.

### Animals

The study was performed on mice and horses. First, mice were used to investigate the safety of vaccine formulation and the immune response caused by different types of adjuvants. Second, the best formulated inactivated AHSV vaccine was applied to horses to quantify antibody responses.

### Horses

The horse farm housed approximately 500 mixed breed horses kept in constant numbers due to a specific herd plan replacement.

Two trials were performed on both adults and young horses (foals). The first was carried out on 15 adult horses, 2–3 years of age. Adult horses underwent one vaccination course with a trivalent live-attenuated AHS vaccine containing AHSV-1, 3, and 4 (LAV) 18 months before the current experiment. The second trial was carried out in 6–7-month-old naïve young horses.

All horses were kept in open paddocks. They were fed concentrated pellet feed (1 kg/100 kg bodyweight) offered twice a day at around 06:00 h and 15:00 h. The pellet feed compositions consisted of 19.5% protein, 5.69% fat, 7.1% fiber, 1.42% calcium, 0.85% phosphorus, and 10.1% moisture content. Roughage, either grass or Pangola hay (1.5 kg/100 kg bodyweight) was offered 3 times a day at about 06.00 h, 15.00 h, and 20.00 h. The horses had unlimited access to water.

### Cell line

MARC-145 cells (Fetal monkey kidney cells, Catalogue No. BSCL 127, Meat Animal Research Center, Nebraska, USA) were cultured in T25 tissue culture flasks with minimum essential medium (MEM) (MEM, Gibco, UK) supplemented with fetal bovine serum (FBS, Gibco, Germany). Cells were passaged twice per week in T75 flasks at a density of 1.5 × 10^6^ cells/mL and incubated in a humidified incubator at 37°C with 5% CO_2_. Viral stocks were propagated in MARC-145 for vaccine production. Vero (green monkey) cells (ATCC, CCL-81™, The American Type Culture Collection, Rockville, Md. USA) were used for immunoperoxidase monolayer assay (IPMA) plate preparation.

### Virus

According to the emerging AHS outbreaks on the horse farm in Thailand, dead horses with AHS clinical signs at QSMI underwent necropsy to determine the cause of the horse’s sudden death. The AHSV serotype 1 was positive in a quantitative polymerase chain reaction (qPCR) from several tissues of the dead horses, including the spleen, lung, tracheobronchial, and mediastinal lymph nodes. For virus isolation, lymph nodes from dead horses were homogenized as a 10% (w/v) suspension in MEM (Gibco, USA) containing 1% penicillin-streptomycin (Sigma-Aldrich, Germany). The suspension was clarified by centrifugation at 3500× *g* for 5 min, and the supernatant was further diluted at 1:10 in MEM. The diluted supernatant was sterile, filtered through a 0.2 μm filter (Sartorius, Germany) and inoculated into MARC-145 cells which maintained in MEM with 5% FBS and 1% penicillin-streptomycin. The culture was incubated at 37°C with 5% CO_2_ until apparency of cytopathogenic effect (CPE) with plaque. Once CPE was observed, the isolated AHS strains were quantitated using qPCR. All collected virus samples containing a sufficient high virus titer were pooled and stored at −80°C as master seed virus [13].

### Vaccine production

Marc-145 cells were incubated with the master seed virus inoculum in MEM with 5% FBS and 1% penicillin-streptomycin at 37°C with 5% CO_2_. The AHSV infectivity titers were calculated before concentration as ≥1 × 10^7^ plaque-forming units/mL. After 7 days of infection, virus particles and cell suspension were harvested by freeze-thawing 3 times. After centrifugation, AHSV was inactivated by the addition of 0.1% formalin (Merck, Germany) and incubated for 2 weeks. The virus was concentrated 10 times and formalin removed using the Labscale™ Tangential Flow Filtration System (Millipore, USA) for prototype vaccine production. The inactivated virus solution was tested for residual viable virus by passaging in MARC-145 cells in T75 tissue culture flasks. The AHSV’s residual viral RNA was detected in the final passages by PCR. All inactivated viral suspensions were stored at 2–8°C until formulation with an adjuvant [14, 15].

### Vaccination design

As a first step, preliminary experiments were carried out in mice using an inactivated AHSV serotype 1 formulated with two different adjuvants to study safety and efficacy in mice before further application in horses. The formulated vaccines were prepared with two different types of adjuvants: Formulated vaccine 1 contained adjuvant 10% Carbigen™ (MVP adjuvants, Nebraska, USA) and formulated vaccine 2 contained adjuvant 10% Gel 01™ (Seppic, Paris, France), to determine the most suitable vaccine in mice. All vaccines were stored at 4–8°C and tested on horses for safety.

### Mouse immunization

Preliminary experiments in mice were performed to select the best adjuvant regarding safety and dosage before choosing immunization in horses. Twenty-five mice were divided into three groups. Group 1 comprised five mice as a control group and was intramuscularly injected with 0.2 mL of phosphate-buffered saline (PBS, pH 7.4) on day 1 and day 22. In Group 2, 10 mice were divided into subgroups I and subgroup II with five mice each, immunized with intramuscular injection of either 0.2 mL or 0.4 mL of formulated inactivated AHSV serotype 1 in 10% Carbigen™, respectively, on day 1 and day 22 on each subgroup. In Group 3, 10 mice were divided into subgroups I and subgroup II with five mice each, immunized with intramuscular injection of 0.2 mL or 0.4 mL of formulated inactivated AHSV serotype 1 with 10% Gel 01™, respectively, on day 1 and day 22 in each subgroup. Blood samples were collected from the tail vein after each immunization were stored in ethylenediaminetetraacetic acid (EDTA) (days 1, 22, and 36) for the determination of seroconversion using IPMA. During the first 36 days after immunization, the clinical signs of each mouse were inspected.

### Vaccination in horses

**Figure-1:**
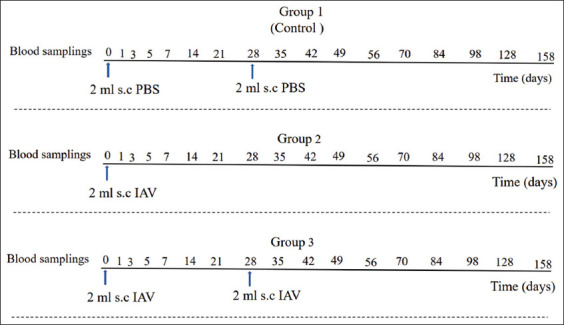
Schematic representation of experimental timeline for measuring antibody titers and physiological parameters (hematology, blood biochemistry, and rectal temperature) response to subcutaneous vaccination (s.c) of formulated inactivated African horse sickness vaccine serotype 1 (IAV) in naïve young horses or adult horses pretreated with the first injection of live-attenuated vaccine serotypes 1, 3, and 4. There are three groups in either naïve young horses or adult horses: Group 1 (control) treated with s.c injection of phosphate-buffered saline on day 0 and day 28, Group 2 treated with s.c injection of IAV on day 0, and Group 3 treated with s.c injection of IAV on day 0 and booster on day 28. Blood samples in ethylenediaminetetraacetic acid were taken from the jugular vein at each specified day for 158 days after vaccination.

Two trials for vaccination of formulated inactivated AHSV serotype 1 in both adult and young horses were carried out, as shown in the experimental protocol in Figure-1. In the first trial, 15 mixed-breed adult horses of both sexes, aged 2–3 years, were selected for the study. Eighteen months before the current experiment, all adult horses had been vaccinated with a LAV vaccine containing AHSV-1, 3, and 4. The horses were divided into three groups of five horses each, the control group and two experimental groups. In Group 1, five horses were subcutaneously injected on day 0 and day 28 with 2 mL of PBS as control. Vaccines in both Groups 2 and 3 were prepared with a freshly formulated inactivated AHSV serotype 1 equivalent of tissue culture infective dose (equivalent to ± 10^7.5^ TCID50/mL) in 10% Gel 01™ for formulated IAV serotype 1. In Group 2, five horses were immunized a single time by subcutaneous injection of 2 mL of IAV on day 0. In Group 3, five horses were immunized twice by subcutaneous injection of 2 mL of IAV on day 0 and day 28. The booster vaccine administered subcutaneously on day 28 was the same as the original vaccine. The formulated IAV was administered in the mid-third of the neck on the left side and boost vaccination on the right side.

EDTA blood samples (20 mL) were obtained from horses on days 0, 1, 2, 3, 5, 7, 14, 21, 28, 35, 42, 49, 56, 70, 84, 98, 128, and 158 after vaccination. On each specified day, blood samples were taken at around 11:00 h after morning feeding to avoid daily variations in activity during the experimental period. Blood samples (whole blood and plasma) were collected from the jugular vein by venipuncture and immediately placed in an ice bath until plasma separation. Before centrifugation, blood samples were analyzed for hematology and blood biochemistry with automated *in vitro* diagnostic medical equipment. The remaining blood was centrifuged at 3500 g for 10 min to separate plasma and stored at −20°C for subsequent analysis of IPMA immunoglobulin G (IgG). The plasma samples from each specified day after IAV vaccination were monitored for the determination of AHSV antigen by qPCR, performed parallel with IPMA IgG analysis. The rectal temperature of each horse was also recorded before blood collection.

In trial 2, twelve 6–7-month-old mixed breed naïve young horses of both sexes were selected for the study. The young horses were divided into three groups with four animals each. The procedures were as in trial 1, including the control group and two experimental groups. Group 1 comprised four horses subcutaneously injected on day 0 and day 28 with 2 mL of PBS as control. In Group 2, four young horses were immunized by subcutaneous injection of 2 mL of IAV on day 0. In Group 3, four young horses were immunized by subcutaneous injection of 2 mL of IAV with boost vaccinations at a 4-week interval on days 0 and 28. Blood samples were obtained from young horses 4 h after morning feeding on days 0, 1, 2, 3, 5, 7, 14, 21, 28, 35, 42, 49, 56, 70, 84, 98, 128, and 158 after vaccination. The EDTA blood sample was divided into portions for antibody measurements, determination of AHSV antigen by qPCR testing, and hematology and blood biochemistry. The rectal temperature of each horse was recorded before blood collection on each blood sampling day.

### Detection and quantification of IgG against AHSV

Specific IgG antibodies against AHSV in sera samples were determined by IPMA. The IPMA plates specific to AHS were prepared as described by Wattanaphansak *et al*. [16] with slight modifications. Briefly, AHSV was inoculated in 1-day-old Vero cells in 96-well plates. The maintained medium (MEM, 2% FBS, 0.5% L-glutamine, and 100 µg gentamicin) was added to coculture with virus inoculums. The plates were incubated at 37°C for 7 days. The supernatant in each well was harvested and 100 µL of 1:1 cold acetone: methanol were added. After 1 min of incubation, the suspension was discarded, and the infected plates were kept at –20°C until further use. For IPMA staining, sera samples were diluted with 5% skim milk in PBS containing 0.05% Tween 20 (PBST) to 1:30, 1:60, 1:120, 1:240, 1:480, and 1:960 concentrations. The 96-well plates containing AHSV were rehydrated with distilled water for 30 min. Each sample (50 µL) was applied to each well and incubated at 37°C for 45 min. After incubation, the suspension was discarded, and the plates were washed 3 times with PBS. The secondary conjugate, horseradish peroxidase rabbit anti-horse IgG (A9292, Sigma, at dilution 1:2,000), was diluted in PBST. Each secondary antibody (50 µL) was added per well and incubated for 45 min. After three washes with PBS, 100 µL of freshly prepared chromogen solution (500 µL of 3-amino-9-ethyl-carbazol, 9.5 µL of acetate buffer, and 5 µL of 30% hydrogen peroxide) was added and incubated at room temperature (28ºC) for 20 min and the solution discards by three washes with distilled water. The dry stained plates were read under an inverted microscope (CKX41, Olympus, Japan) to determine the final antibodies titer.

### Hematology, plasma biochemistry, and body temperature

Hematology, blood biochemistry, and body temperature were calculated to assess the effect of IAV vaccination. Blood samples were obtained from horses on each specified day, on day 0 before vaccination, and on subsequent days up to 42 days after vaccination. Part of the anticoagulated blood was used for hematology analysis. Various hematological parameters, namely, total erythrocyte count (red blood cells), packed cell volume, hemoglobin, total leukocyte count, white blood count (WBC), and white cell differential counts for neutrophils, monocytes, lymphocytes, eosinophils, and basophils were determined using an automated hematology analyzer (IDEXX ProCyte Dx, Westbrook, USA). Blood biochemistries were measured with a Vet Test Chemistry Analyzer (IDEXX, UK). Body temperature was measured between 11.00 and 12.00 on each specified day using a digital thermometer through rectal temperature.

### Statistical analysis

All measurement data of hematology, blood biochemistry, and rectal temperature are shown as the means ± standard deviation (SD). The paired t-test was used to estimate significant differences between values obtained from day 0 (control) and each specified day after vaccination in the same group. p < 0.05 was considered to indicate statistical significance.

## Results

### Immunogenicity of inactivated AHSV formulated with different adjuvants in mice

Three weeks after initial immunization with 0.2 mL PBS and boost on day 21, mice in Group 1 remained negative for IPMA IgG on 22 days post-vaccination and 36 days after boost immunization. In Group 2, one out of five mice receiving 0.2 mL of formulated inactivated AHSV antigen in 10% Carbigen™ was positive for IPMA IgG at 1:60, 1:120, and 1:30 on day 14, day 22, and day 36 after vaccination, respectively. Among mice immunized with 0.4 mL of formulated AHSV antigen in 10% Carbigen™ on day 0 and boosted on day 21, two out of five mice were positive for IPMA IgG at 1:60, 1:30, and 1:30 on day 14, day 22, and day 36, respectively. Interestingly, two out of five mice receiving 0.2 mL of formulated AHSV antigen in 10% Gel 01™ (Group 3) were positive for IPMA IgG (endpoint) at 1:30–1:120, 1:60–1:120, and 1:60 on day 14, day 22, and day 36, respectively. Moreover, increasing formulated AHSV antigen concentration (from 0.2 to 0.4 mL) in 10% Gel 01™ could increase the number of positive mice and the seroconversion level. Four out of five mice were positive for IgG against AHSV at 1:60–1:120 on day 14, 1:30–1:60 on day 22, and 1:30–1:120 on day 36, respectively. Based on these results, the formulated AHSV antigen in 10% Gel 01™ was the most promising adjuvant in terms of safety and dosage to formulate IAVs.

### Immunogenicity with formulated IAV serotype 1 in adults and naïve horses

**Figure-2:**
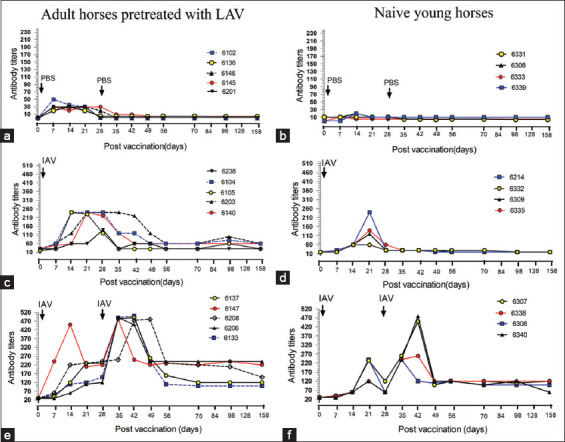
Effects of vaccination with inactivated African horse sickness (AHS) virus serotype 1 (IAV) formulated with 10% Gel 01™ (IAV) on antibody titers (immunoperoxidase monolayer assay-immunoglobulin G [IPMA-IgG]). Horses in each group were subcutaneously injected with phosphate-buffered saline or IAV vaccine on day 0 and day 28 (indicated arrow). (a) The antibody titers were analyzed on each specified day in adult horses in Group 1, (b) young horses in Group 1, (c) adult horses in Group 2, (d) young horses in Group 2, (e) adult horses in Group 3, and (f) young horses in Group 3. Y-axis shows antibody titers according to the level of seroconversion IPMA-IgG (endpoint).

The effects of subcutaneous vaccination of formulated IAV with 10% Gel 01™ (Seppic, France) on antibody responses were performed in three groups of horses in trial 1 and trial 2 (Figure-2).

In Group 1, the IPMA IgG levels of unvaccinated adult horses and naïve young horses remained negative after subcutaneous injection with PBS (Figures-2a and b); however, low antibody titers (1:30) were still detected in some adult horses pretreated with LAV about 18 months before the present study.

In Group 2, all horses showed seroconversion for 2 weeks and more increased antibody titers in adult horses. The peak of antibody titers of four out of five horses maximally increased (1:240), having a more prolonged peak of antibody titers, approximately from day 14 to 36 followed by a gradual decline to control levels from day 42 to 56 after vaccination (Figure-2c). In comparison to naïve young horses in Group 2 in trial 2, the prime vaccination with IAV showed a positive response to an increase in antibody titers on day 14 in all young horses but one out of four young horses showed an increase in high antibody titer close to 1:240 on day 21. After a transient increase in antibody titers occurred for 21 days, a gradual reduction in antibody titers occurred to the initial level on day 28 after vaccination (Figure-2d).

In Group 3, both adult horses and naïve young horses showed an increase in antibody titers after initial vaccination with IAV on day 0. The highest titers for adult and young horses were 1:480 on day 14 and 1:240 on day 21, respectively. These antibody titers apparently decreased on day 28 before boost vaccination. After boosting on day 28, the highest antibody titers (1:480) were detected between days 36 and 42 in adult horses, while the highest antibody titers of 1:480 were detected on day 42 in two young naïve horses. The high antibody titers in all horses decreased on days 49–56 for adult horses and on day 49 for young horses. The antibody titers of three out of five adult horses decreased to 1:240 on day 49, while in the other two adult horses decreased to 1:120 on day 56. These antibody titer levels were sustained throughout the study period (Figure-2e). This pattern of antibody titers differed from young horses, in that four horses showed decreasing titers to 1:120 on day 49 after vaccination and were sustained throughout the study (Figure-2f). In conclusion, IAV with 10% Gel 01™ adjuvant might be the most promising formulation, although improvement by minimizing adverse effects and increase of the mean antibody titers in young horses and further study for serum neutralization tests are needed to guarantee safety, efficacy, and protection.

However, EDTA plasma samples from each specified day after IAV vaccination in both naïve young and adult horses were monitored for the determination of AHSV antigen by qPCR. The negative qPCR results revealed no virus in the plasma each day up to 158 days post-vaccination (data not shown).

### Changes in body temperatures and local reactions after vaccination

**Figure-3:**
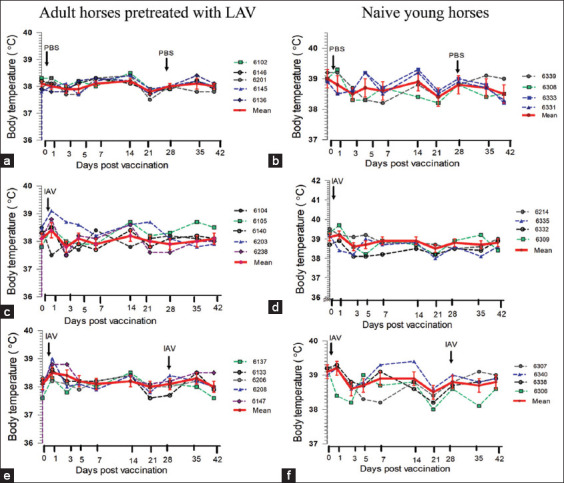
Effects of vaccination with inactivated African horse sickness virus serotype 1 (IAV) formulated with 10% Gel 01™ on body temperature. Horses have subcutaneously injected with phosphate-buffered saline or IAV vaccine on day 0 and day 28 (indicated arrow). The rectal temperatures were measured between 11:00 and 12:00 h of each specified day in adult horses in Group 1 (a), young horses in Group 1 (b), adult horses in Group 2 (c), young horses in Group 2 (d), adult horses in Group 3 (e), and young horses in Group 3 (f). The red line in each group represents the mean values of rectal temperature (mean ± standard deviation).

The mean body temperature in young and adult horses during IAV vaccinations did not differ from those of controls for 42 days post-vaccination(Figures-3a-f). On day 1 post-vaccination, a slight elevation of rectal temperature was found in some horses, particularly in the afternoon; however, no significant difference was noted with respect to that on day 0. No significant changes in any other clinical signs were recorded over the observation time, including no skin thickening at the site of IAV injection. The variations in estimated rectal temperatures would relate to environmental temperatures, lower in the morning and peaking in the afternoon. Rectal temperatures remained in the normal range for horses, from 37.2°C to 38.9°C.

### Changes in hematological values in horses vaccinated with IAV

**Figure-4:**
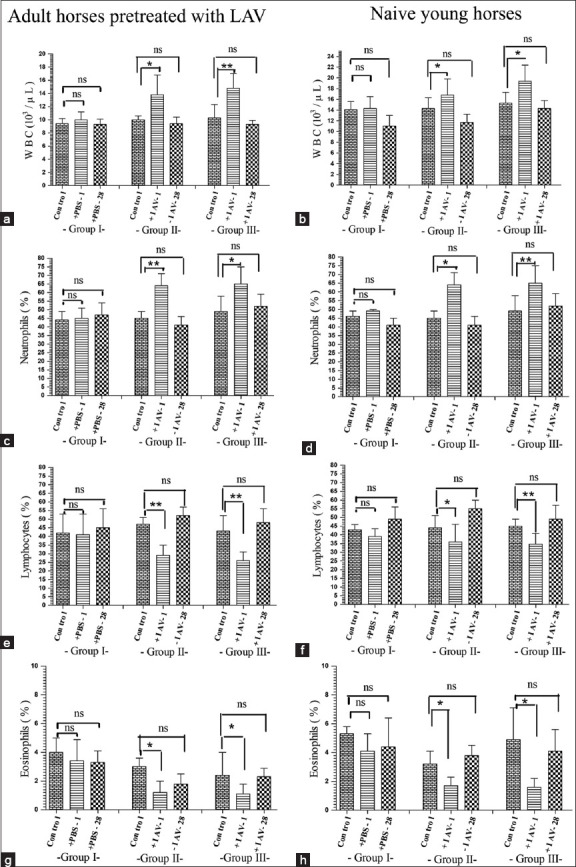
Effects of subcutaneous injection of formulated inactivated African horse sickness virus serotype 1 (IAV) on the changes in total white blood cell count (a, b), and differential counts [neutrophils (c, d), lymphocytes (e, f), and eosinophils (g, h)] on day 1 (+IAV1) and day 28 (±IAV28) after vaccination in three groups of either adult horses pretreated with live-attenuated vaccine or naïve young horses. Group 1 was control that received phosphate-buffered saline. Group 2 vaccinated with IAV on day 0. Group 3 vaccinated with IAV on day 0 and 28. ns=Non-significant; the significant difference by paired t-test with control (*p < 0.05 and **p < 0.01).

All hematological values (mean **±** SD) were measured on day 0 (control) and various time points after vaccination on day 1–day 42 in adult horses and naïve young horses. In addition, Figure-4 shows that after initial IAV vaccination on day 0, in Groups 2 and 3 of both naïve young and adult horses pretreated with LAV vaccine, the total WBC and white cell differential counts for neutrophils on day 1 were significantly higher (p < 0.05) than those of the control on day 0, while white cell differential counts for lymphocytes and eosinophils significantly decreased (p < 0.05) on day 1 after IAV vaccination in both groups in adult and naïve young horses. These values returned to control values on day 0 within the next 2–3 days after vaccination. In addition, no other hematological parameters differed between the control on day 0 and other days after vaccination over the 42 days of study (S1 and S2). There were no alterations in any hematological parameters from day 0 to day 42 in animals receiving PBS injections on day 0 and day 28.

### Biochemical parameters in horses vaccinated with IAV

According to the present study, a more comprehensive study of blood biochemistry can help understand significant adverse effects after vaccination. All plasma biochemical and electrolyte parameters of adult and young horses revealed no significant differences between day 0 and day 30 after vaccination. The glucose, creatinine, urea, phosphorus, calcium, total protein, albumin, globulin, aspartate aminotransferase, gamma-glutamyltransferase, and alkaline phosphatase plasma concentrations were within normal limits.

## Discussion

In the present study, to develop inactivated AHS vaccines, inactivated AHSV serotype 1 formulated with appropriated adjuvants was studied as a prototype vaccine on efficacy and safety concerning local reactions. The best available AHS vaccine is needed to induce the neutralizing antibody response in protection. However, many studies revealed a possible evidence concern of multivalent LAVs, which is not safe concerning residual virulence, reverse virulence, reassortment, and subsequently leading to virus spread by midges, reproduction losses, and virulent AHSV variants [1, 9]. These effects were examined and compared with those of studies on the use of attenuated vaccines of bluetongue virus (BTV), a prototype of the genus Orbivirus, which morphological characteristics are identical to those of AHSV, which was not recommended and the recent BTV outbreak in Europe was controlled using inactivated vaccines [17, 18]. Furthermore, the use of monovalent AHS vaccines is beneficial and effective against only one serotype of the infecting strain, while polyvalent vaccines are effective against ≥2 serotypes of the virus causing the outbreak [19]. Polyvalent AHS vaccines significantly reduce the impact of AHS, despite outbreaks even in well-vaccinated horses; the vaccine itself has occasionally caused disease in inoculated horses [20, 21]. Moreover, a case of polyvalent AHS vaccine failure in a 5-year-old polo mare has been noted [22].These disadvantages hamper disease control and safe movement and trade of LAV vaccinated horses. Therefore, the choice of AHS vaccine types is important, particularly the use of multivalent LAVs in areas without prior AHS outbreaks.

The Livestock Department attempts to control AHS as an emerging disease using available live-attenuated AHS vaccine serotypes 1, 3, and 4, although several reports indicated that IAV should be a choice to use in a regimen for immunization in the horse in an area where no current outbreaks of AHS were before. In the present study, we developed formulated inactivated AHSV serotype 1 (IAV) with 10% Gel 01™ as an adjuvant as a prototype vaccine and evaluated its efficacy and safety.

The present vaccination was performed by subcutaneous injection of 2 mL IAV formulated in 10% Gel 01™. This formulated IAV did not induce local reactions at the site of vaccination in either adult or naïve young horses; these remained invisible throughout the 158-day experimental period. However, rectal temperature slightly increased for 1 day after vaccination compared to day 0, in 2/5 adult horses (Figure-3). There were no alterations in hematological values over 42 days in control horses receiving PBS injections on day 0 and day 28. In both young and adult horses receiving the formulated IAV, the leukocytes count and %neutrophils count on day 1 were significant higher (p < 0.05) than those of the control on day 0, while %lymphocytes and eosinophil count significantly decreased on day 1 after IAV vaccination. These values returned to control values on day 0 within the next 2–3 days. These changes in white blood cells could be attributed to a bodily response to the first vaccination; especially, lymphopenia would probably be a response to stress and the viral load in the vaccine [23]. These results do not imply IAV-induced viremia, since the plasma samples of both naïve young horses and adult horses on each day up to 158 days after IAV vaccination were negative in qPCR testing for the AHSV antigen. However, other mechanisms, for example, stress, failure to rest, and improper timing for vaccination, should be considered [24].

Clearly, the mild local reactions in both young horses and adult horses, resulting from inactivated AHSV serotype 1 using 10% Gel 01™ as adjuvant differed from the previous reports [25], showing adverse effects and inflammatory local reactions after vaccination with AHS comparing intramuscular vaccination with subcutaneous vaccination of formulated IAV with water based adjuvant gel and immune stimulating complex as adjuvants, indicating that local reactions by intramuscular vaccinations were less than by subcutaneous vaccinations for both formulations. Subcutaneous vaccination rout can induce temporary or long-term local reactions in some horses; this safety aspect is extremely important for the equestrian industry and owners of pet horses. The differing results might arise from different formulations and adjuvant types. In the present study, cell culture AHSV serotype 1 was produced on monolayers of Vero cells by serial refreshing of culture medium, and harvests of culture medium were pooled and used as AHSV antigen. Culture medium harvests of cell culture before cytopathogenic effect (CPE) contained >10^7^ TCID50/mL AHSV serotype 1. Pooled harvests are free of cell components and free of non-structural AHSV proteins. Costly down processing steps, such as purification and concentration of produced AHSV antigen [26], were not needed.

We compared the immune responses to the administration of formulated IAV between naïve young and adult horses pre-immunization with polyvalent LAV serotypes 1, 3, and 4.

In this study, the similar vaccination route and amount of antigen and adjuvant in the IAV preparation used in the present study showed fast increases in antibody titers in adult horses pretreated with LAV vaccines in contrast to naïve young horses with a primary IAV injection, despite a correlation between the amount of antigen and antibody response [27]. These results may be explained by a mechanism involving the adaptive immune system. The B lymphocytes of adult horses would form memory B cells that “remember” the first LAV injection (containing serotypes 1, 3, and 4). The AHSV serotype 1 in the formulated IAV vaccine in the present study may induce faster antibody production in the second induction, even though memory B cells circulate in a quiescent state in the bloodstream and low antibody titers were found on day 0 in the adult horses in the present experiment. Memory B cell’s function is to memorize the characteristics of the antigen that activated their parent B cell during the initial infection such that if the memory B cell later encounters the same antigen, it triggers an accelerated and robust secondary immune response. Memory B cells have B-cell receptors on their cell membrane, identical to the one on their parent cell, that allows them to recognize AHSV serotype 1 and mount a specific antibody response [28]. Thus, the primary injection of formulated IAV vaccine in adult horses immunized with a LAV vaccine would induce rapid high antibody titers than those of naïve horses with the primary injection of formulated IAV vaccine. However, antibody titers of adult horses were immunized by one injection of LAV vaccines remained low (1:30–1:60) at 18 months after immunization (Figure-2a). Our results showed dissimilarity antibody responses between horses pre-vaccinated with the LAV vaccine and naïve young vaccinated horses. The high antibodies titers responses remained high 1–2 months after the first injection in adult horses suggest that the fast responses in adult horses pretreated with the LAV vaccine. This may be explained, at least in part, by immune responses and host factors such as age and health but not the nature of the vaccine itself could explain this effect. However, a single IAV serotype is necessary for controlling outbreaks where the specific AHSV serotype is known. Importantly, naïve horses vaccinated with formulated IAV serotype 1 after boosting on day 28 developed an appropriate high antibody titer after a second booster. Under current circumstances of the short immune responses, it needs revaccination of the third booster of IAV vaccine after 6 months of vaccination within a year. The present results would support a previous study showing that horses vaccinated with single serotypes need a booster after 6 months and simultaneously immunized horses need it after 12 months [29], even if inactivated *Orbivirus* vaccines are safe, as the virus does not revert to virulence or cause viremia in vaccinated animals or reassort with field *Orbivirus* strains [30]. However, recent research has shown that multiple vaccinations with IAV containing all nine serotypes did not prevent AHSV in approximately 10% of vaccinated horses despite high neutralizing antibodies [31].

## Conclusion

The present study indicates that administration of an IAV serotype 1 formulation using 10% Gel 01™ as an adjuvant is safe and induces a high antibody response in horses without local reaction and mild systemic effects in both naïve young and adult horses pre-immunized with LAV. The present study suggests that to achieve high immune responses in young vaccinated horses with formulated IAV serotype 1, a third dose IAV vaccine at 6 months of vaccination is needed. Naïve horses required ≥2 vaccinations and annual booster vaccination to achieve high antibody titers. To better understand the immune response and develop more effective and safer vaccines, further research on better adjuvants for use in horses is needed to develop safe, efficacious, and acceptable AIS for horse owners.

## Data Availability

The supplementary data can be available from the corresponding author on a reasonable request.

## Authors’ Contributions

NC, SW, RT, SA, SO, and DN: Contributed to the conception and designed the study. NC, SW, RT, and SA: Contributed reagents, materials, and analytic tools. SW, SA, SO, and DN: Performed the animal experiments. NC, SW, and SO: Statistical analysis and interpretation. NC and SW: Wrote and revised the paper. All authors have read and approved the final manuscript.
